# Improving the design of observational studies in orthopaedics: a practical guide to target trial emulation

**DOI:** 10.1007/s00264-026-06897-x

**Published:** 2026-06-13

**Authors:** Masaki Hatano, Yuya Kimura, Hideo Yasunaga, Sakae Tanaka

**Affiliations:** 1 Department of Orthopaedic Surgery, Faculty of Medicine, The University of Tokyo, Tokyo, Japan; 2 Department of Health Services Research, Graduate School of Medicine, The University of Tokyo, Tokyo, Japan; 3 Department of Clinical Epidemiology and Health Economics, School of Public Health, The University of Tokyo, Tokyo, Japan

**Keywords:** Target trial emulation, Orthopaedics, Observational study, Hip fracture, Epidemiology, Causal inference

## Abstract

**Purpose:**

To explain how target trial emulation can help orthopaedic researchers design observational studies that are more clinically interpretable and less vulnerable to avoidable design bias.

**Methods:**

We provide a methodological overview of target trial emulation for orthopaedic research, using the comparison of postoperative outcomes after total hip arthroplasty versus bipolar hemiarthroplasty for hip fracture as an illustrative example. The review focused on translating a causal question into a target trial protocol, how conventional observational designs can introduce bias, and how routinely collected data can be used to emulate a target trial when key design conditions are met.

**Results:**

Target trial emulation begins by specifying the clinical question, hypothetical trial, and assumptions required for causal interpretation. This explicit specification is particularly relevant to orthopaedic observational research, wherein treatment decisions are often influenced by prognosis, functional status, surgeon judgment, and institutional context. Conventional observational analyses may be difficult to interpret when the eligibility criteria include patients for whom one treatment is not realistic, treatment labels combine important procedural variations, treatment assignment and follow-up are not aligned at time zero, or post-baseline information is used to modify follow-up or exclude patients. This framework can emphasise these problems before analysis, although it cannot overcome residual confounding, poor measurement, informative loss to follow-up, or other data limitations.

**Conclusions:**

Target trial emulation can guide observational studies’ design around treatment decisions encountered in routine practice. Its application may help orthopaedic researchers produce studies that are more transparent, clinically interpretable, and better aligned with decision-making in practice.

## Introduction

Musculoskeletal disorders often have multiple treatment options for the same clinical condition. Therefore, clinical research in this field is predominantly focused on comparing the benefits and risks of these alternatives. Although randomised controlled trials are the gold standard for evaluating treatments, many key questions in musculoskeletal disorders cannot easily be addressed through these trials. Feasibility in surgical research may be constrained by ethical considerations, strong treatment preferences, challenges in blinding, variation in technical expertise, cost, and need for extended follow-up. Consequently, observational data sources have become increasingly important for evaluating orthopaedic interventions [1].

The increasing accessibility of administrative claims data, national registries, and electronic health records has generated new prospects for observational research in orthopaedic surgery. These data sources support the study of interventions used in routine practice and address questions that are difficult to examine in randomised controlled trials. However, the main challenge of observational research extends beyond the absence of randomisation and the need to adjust for imbalances in measured baseline characteristics. A fundamental problem is that causal questions are often not clearly defined at the design stage. When the causal question remains implicit, the resulting analysis may investigate an ill-defined clinical question and yield difficult-to-interpret or difficult-to-apply findings [2].

This problem is particularly important in orthopaedic research because causal inference from observational data can be undermined by confounding and inadequate translation of the clinical question into study design. In routine practice, operative decisions depend on prognosis, functional status, surgeon judgment, and institutional resources. When these decisions are reconstructed retrospectively from routine data, the analysis may fail to reflect a clearly defined intervention, population, or a clinically meaningful time point. In such circumstances, bias can arise not only from confounding by indication but also from misalignment of eligibility, treatment assignment, and the start of outcome follow-up.

The target trial emulation framework addresses this problem by requiring investigators to specify the trial they aim to emulate before analysing the observational data [3, 4]. Instead of starting with an available dataset and shaping the analysis around it, this framework requires investigators to first translate the clinical question into a causal question, specified as a hypothetical pragmatic target trial, and then evaluate how closely it can be emulated using observational data. The framework's practical value not lies not in making observational data equivalent to randomised data but rather in requiring investigators to define a clinically meaningful intervention, an appropriate comparison, a defensible time zero, and an estimand (i.e., the specific treatment effect that the study aims to estimate, aligned with the clinical question of interest) [3, 4]. Time zero refers to the clinical point at which eligibility is assessed, treatment strategies are assigned, and outcome follow-up is initiated.

Although target trial emulation has garnered attention in clinical epidemiology, many orthopaedic surgeons, clinical researchers, and policymakers may be unfamiliar with its rationale, terminology, and practical implications. A better understanding of this framework could improve the design of observational studies and help interpret their findings in orthopaedic surgery. In this review, we used the comparison of postoperative outcomes after total hip arthroplasty (THA) versus bipolar hemiarthroplasty (BHA) for hip fracture using a large healthcare claims database as an example to address the following three practical questions: (i) How can orthopaedic clinical questions be translated into target trials? (ii) Why do conventional observational analyses often fail to provide credible answers? and (iii) How can target trial emulation be applied using routinely collected data?

## From orthopaedic clinical questions to target trials

### Defining the clinical question before analysing observational data

When investigators use observational data to determine how a treatment may affect subsequent outcomes, a useful starting point is to consider the randomised trial that would best tackle the clinical question. The target trial emulation framework follows this logic. Rather than beginning with the available database and asking which analysis can be performed, investigators first clarify the treatment effect that they seek to estimate and then assess how closely the corresponding target trial can be emulated using non-randomised data. For orthopaedic researchers, this means starting with a real clinical question, such as the risk of two year revision surgery after THA versus BHA for hip fracture or postoperative complications and mortality after surgery within 24 h versus after 24 h of hip fracture, rather than with the variables available in a registry or claims database.

### Defining the causal estimand

After formulating the clinical question, investigators should define the causal estimand before choosing an estimator. The causal estimand is the treatment effect that the study aims to estimate at the population level, while the estimator is the analytic procedure used to estimate that quantity from data. In the target trial framework, the causal estimand is made explicit by specifying the key components of the hypothetical trial, viz. eligibility criteria, treatment strategies, treatment assignment, outcomes, follow-up commencement and cessation, and causal contrasts of interest [3]. These components specify the effect that is being estimated, in whom, under which treatment strategies, over what period, and which causal contrast is used.

For example, in a study comparing THA with BHA for hip fractures, the causal estimand may be defined as the intention-to-treat average treatment effect of assignment to an initial THA strategy versus assignment to an initial BHA strategy on two year revision surgery among eligible patients with femoral neck fracture. In this example, the estimand specifies the target population, treatment strategies, outcomes, follow-up periods, and causal contrast. However, at this stage, the estimand does not need to list every operational detail. These details can be specified separately in the target trial protocol, as illustrated in Table 1. The estimator is the analytic procedure used to estimate this effect from observational data, such as inverse probability of treatment weighting combined with a regression or survival model. Thus, the estimand defines the causal quantity of interest, whereas the estimator specifies how that quantity is estimated in the available data.

**Table 1:** Pragmatic target trial specification and observational emulation for comparing 2-year revision surgery after THA versus BHA for hip fracture

Protocol component	Target pragmatic trial specification	Target trial emulation
Eligibility criteria	**Inclusion Criteria** 1. Adult aged 60 years and above with fracture of the femoral neck 2. Operative treatment within 3 days (i.e., 72 h) after initial admission 3. Patient was ambulatory prior to fracture, although they may have used an aid such as a cane or walker 4. No other major trauma 5. Treatment at a hospital performing at least 50 THA or BHA procedures annually **Exclusion Criteria** 1. Patient not suitable for BHA (i.e., inflammatory arthritis, rheumatoid arthritis, pathologic fractures, or severe osteoarthritis of the hip) 2. Associated major injuries of the lower extremity (i.e., ipsilateral or contralateral fractures of the foot, ankle, tibia, fibula, knee, or femur; dislocations of the ankle, knee, or hip; or femoral head defects or fracture) 3. Infection around the hip (soft tissue or bone) 4. Patients with a disorder of bone metabolism other than osteoporosis (i.e., Paget’s disease, renal osteodystrophy, osteomalacia) 5. Patients with Parkinson’s disease or dementia 6. Patients transferred from another hospital or healthcare facility after initial admission for the index fracture	Same as the target trial, except that pre-fracture ambulatory status could not be directly ascertained in the database
Treatment strategies	Participants would be assigned to undergo either THA or BHA for femoral neck fracture. Both strategies would be delivered at hospitals performing at least 50 THA or BHA procedures annually Both THA and BHA would be delivered within a common perioperative care framework. Antibiotic prophylaxis, thromboprophylaxis, and postoperative mobilization with weightbearing as tolerated would be recommended for both groups, according to contemporary local protocols For THA, the treatment strategy would consist of arthroplasty performed according to usual practice. To preserve feasibility and pragmatism, the trial would not standardize the surgical approach, use of cemented components, or implant manufacturer For BHA, surgeons would be required to use modern BHA implants. Non-modular, non-canal-filling unipolar implants (such as Moore’s and Thompson’s prostheses) would not be permitted	Same as the target trial, with treatment strategies operationalized using procedure codes and available device information in the database
Treatment assignment	Individuals would be randomly assigned to THA or BHA. Owing to the nature of the interventions, participants would be aware of the treatment strategy they received	Individuals were classified according to the treatment strategy initiated at time zero (THA or BHA). To emulate random treatment assignment conditional on the measured baseline covariates, inverse probability of treatment weighting was used. Time zero was defined as the date of the index arthroplasty procedure
Follow-up period	The follow-up period begins at the initiation of the treatment strategy and ends at the earliest occurrence of one of the following events: the study endpoint, 2 years after the index date, death, loss of follow-up, or the end of the study period	Same as the target trial, except that loss to follow-up was operationalized as disenrollment from the database and end of follow-up was defined using information available in the database
Outcomes	Revision surgery within 2 years of initial surgery to promote fracture healing, relieve pain, treat infection, treat a peri-prosthetic fracture, or improve function. It includes the following: 1. Reduction of hip dislocation 2. Revision surgery with another implant 3. Incision and drainage for any infection 4. Implant removal	Same as the target trial, except that revision surgery was operationalized using procedure codes recorded in the database
Causal contrasts of interest	Intention-to-treat average treatment effect	Observational analogue of intention-to-treat average treatment effect
Statistical analysis	Intention-to-treat analysis	Observational analogue of intention-to-treat analysis
Identifying assumptions	Randomisation ensures baseline exchangeability between treatment groups. Additional identifying assumptions include consistency, no interference between individuals, and complete follow-up or non-informative censoring. If censoring occurs, valid estimation requires conditional exchangeability and positivity for censoring, given measured predictors of censoring	Conditional exchangeability for treatment assignment given measured baseline covariates, positivity of treatment assignment conditional on those covariates, consistency, and no interference between individuals. If censoring occurs, estimation also requires conditional exchangeability and positivity for censoring, given measured predictors of censoring

*THA* total hip arthroplasty, *BHA* bipolar hemiarthroplasty

### Specifying the target trial protocol

**Fig. 1:**
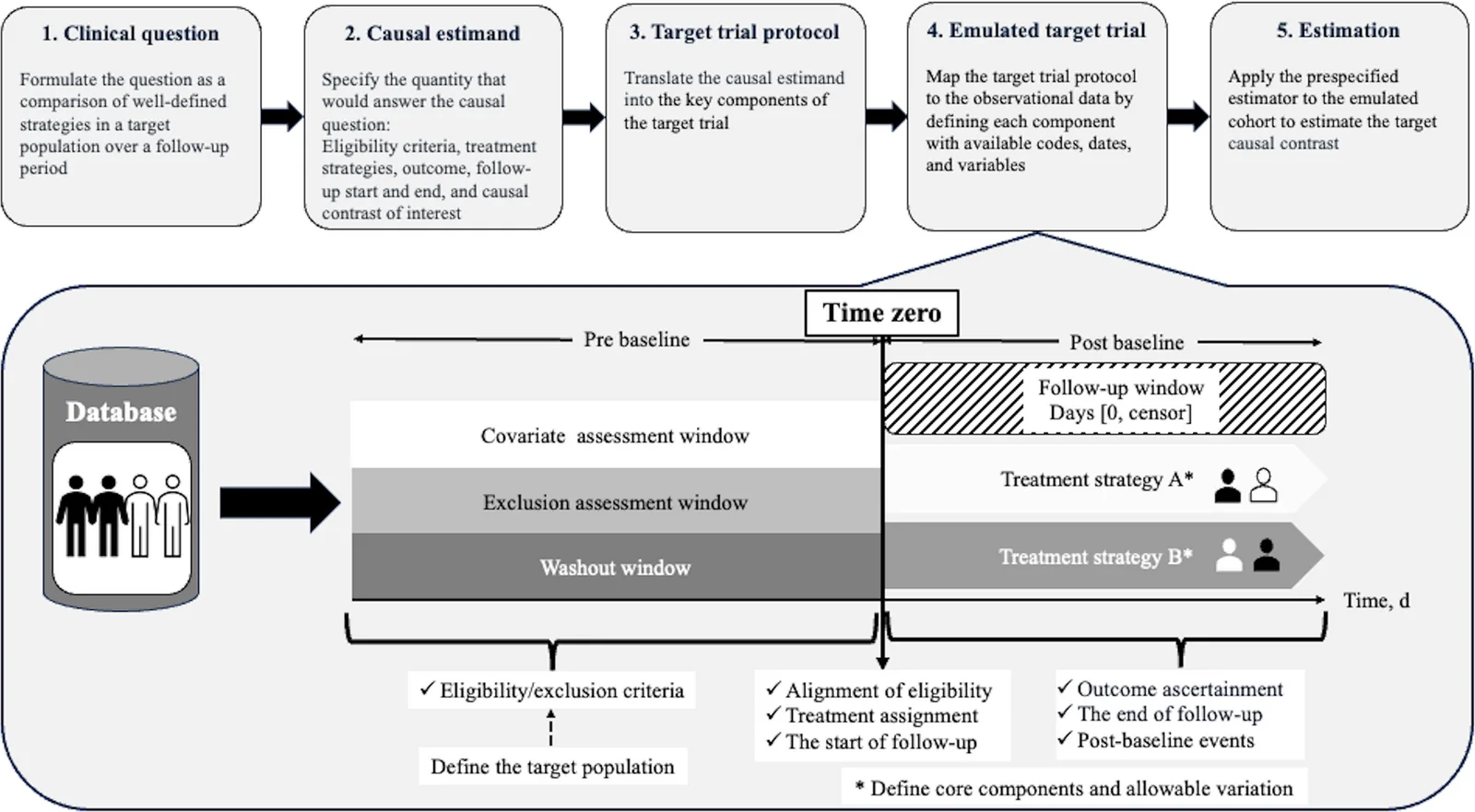
Implementing the target trial emulation framework in orthopaedic research

The target trial protocol provides the structure for operationalising this estimand in observational data (Fig. 1) [3]. This component specifies how the causal estimand will be estimated in the emulation under the requisite identification and modelling assumptions [3, 4]. A central feature of this process is the alignment of eligibility, treatment assignment, and commencement of outcome follow-up at time zero. Making these elements explicit gives the research question a clearer operational definition and attenuates the risk of the vague comparisons that often arise in conventional observational analyses.

Because the emulation of a target trial using observational data does not include a randomised treatment assignment, the assumptions required for causal interpretation should be stated separately from the protocol components, as described below.

### What the framework can and cannot address

A major strength of this framework is that it reduces ambiguity in study design. By requiring investigators to specify the intended trial before conducting the analysis, design flaws are easier to identify before they become embedded in the analysis. However, although target trial emulation can improve the study design, it cannot compensate for inadequate data [3]. Residual confounding, measurement errors in treatment or outcome definitions, incomplete information on clinically important covariates, and informative loss to follow-up may threaten the validity of the results. Thus, target trial emulation should not be deemed sufficient to justify causal interpretation. Rather, it helps distinguish avoidable design problems arising from limitations persistent in the data.

### Core identifying assumptions

An observational study using this framework should describe how the target trial was emulated and what assumptions are required to support a causal interpretation. The most important assumptions are consistency, conditional exchangeability, and positivity [5]. For orthopaedic readers, these assumptions can be understood in the following clinical terms: the procedures under comparison must be sufficiently well-defined, patients in the treatment groups must be comparable after adjustment for measured pretreatment factors, and both strategies must be realistic options within the target population.

## Design pitfalls in orthopaedic observational research

Observational studies are prone to several biases that can undermine causal interpretation [6]. In this section, we illustrate these biases using observational comparisons of the postoperative outcomes in patients undergoing BHA or THA for hip fracture.

### Ill-defined eligibility criteria, estimands, and lack of positivity

In observational surgical research, treatment selection is often influenced by prognostic characteristics. Confounding by indication can arise when these characteristics are also associated with postoperative outcomes. For example, among patients with hip fracture, those with poor pre-fracture function, limited independence in activities of daily living, or a high burden of preoperative comorbidities may be considered less suitable for THA in routine practice and therefore more likely undergo BHA [7–10]. If the baseline prognosis is imbalanced between the groups in this way, the BHA group may appear to have a higher risk of postoperative medical complications. Thus, the observed differences may reflect baseline differences in prognosis rather than a true difference in the comparative efficacy of BHA and THA.

Fundamentally, this problem extends beyond the covariate adjustment and includes the definitions of the target population and estimand [5]. For example, if a study includes many patients for whom THA was never a realistic treatment option, the estimated effect may not reflect the estimand relevant to surgical decision-making. In some patient characteristic strata, the probability of receiving THA may be close to zero. This raises concerns about violation of the positivity assumption, which weakens the clinical interpretability of the estimated effect [5].

### Imprecisely defined treatment strategies

When treatment strategies are ambiguously defined, the consistency assumption becomes less plausible, and the causal effect of interest may not be clearly interpretable [5]. In orthopaedic surgery, this problem is relevant in studies using routinely collected data where treatment groups may differ not only in patient prognosis but also in procedural and service-level factors, including implant design, fixation method, surgical approach, surgeon experience or case volume, hospital volume, and perioperative care pathways [11–13]. In the comparison of BHA versus THA for hip fracture, differences in outcomes may therefore reflect these co-interventions and procedural versions rather than the effect of the arthroplasty strategy itself.

### Misalignment of eligibility assessment, treatment assignment, and the commencement of outcome follow-up (at time zero)

In many observational analyses, a faulty temporal alignment is a more serious problem than incomplete adjustments. Target trial emulation emphasises that eligibility, treatment assignment, and the start of follow-up should be aligned at time zero [4]. If these elements are not synchronised, the analysis may introduce avoidable design problems, including immortal time bias and selection-related distortions.

**Fig. 2:**
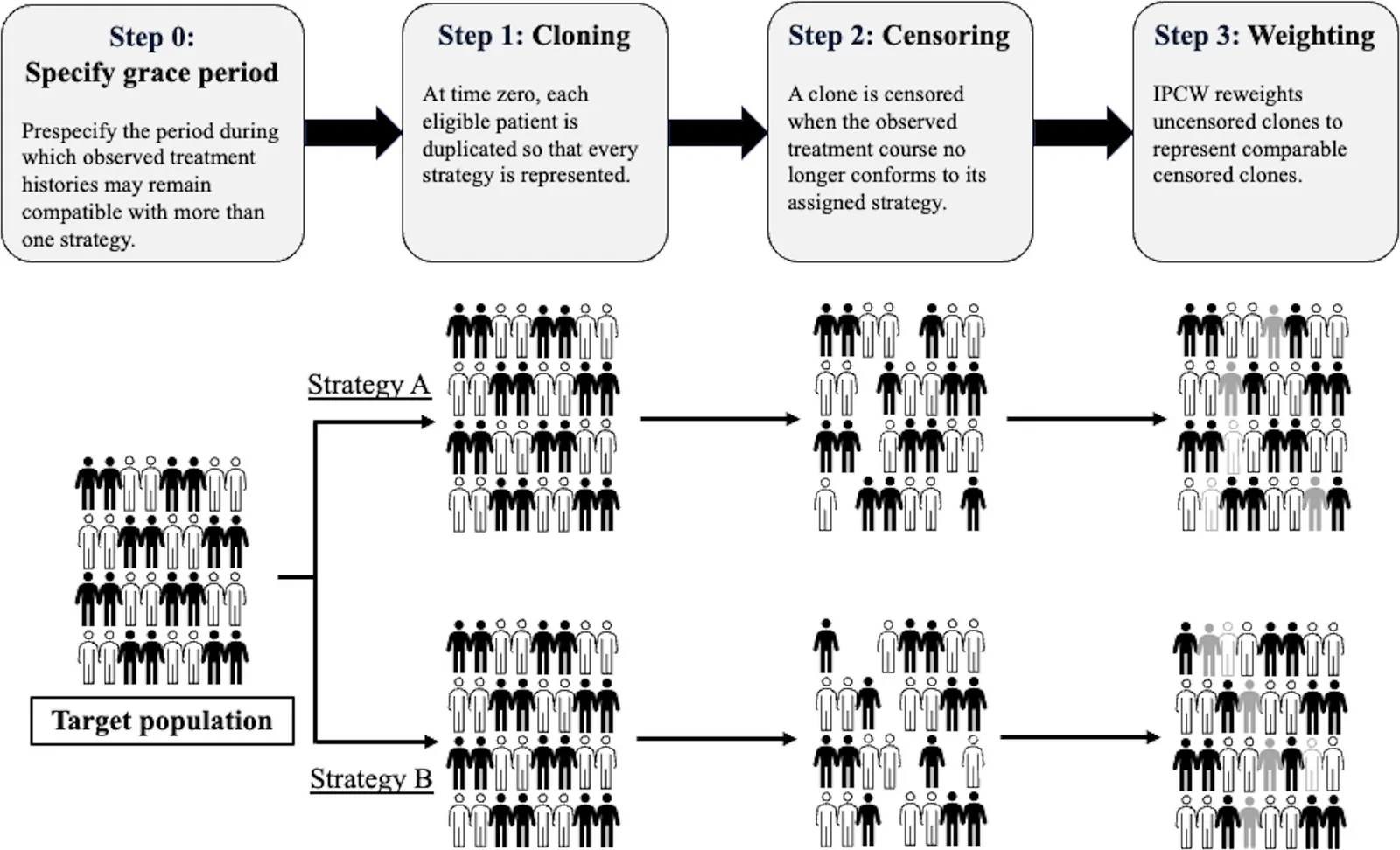
Illustration of clone-censor-weighting in target trial emulation. IPCW: inverse probability of censoring weight

This issue is important in orthopaedic research because many clinically relevant questions involve the timing of treatment. An example is the postoperative complications and mortality after early versus delayed surgery for hip fracture. This question should not be addressed by retrospectively classifying patients according to the surgery time observed after cohort entry. A target trial would define the timing strategies at clinical presentation, such as hip fracture surgery within 24 h versus after 24 h, and would start follow-up at that same point. For such timing strategies, clone-censor-weighting can be used when the compatibility with each strategy is determined over a pre-specified grace period. Each eligible patient is cloned at time zero, and the clones are analytically allocated to each strategy. The clones are artificially censored when the observed treatment history becomes incompatible with the assigned strategy. The inverse probability of censoring weights accounts for the informative censoring introduced by artificial censoring, under the required assumptions. The weighted analysis estimates the per-protocol effect targeted by the emulated trial (Fig. 2). Sequential target trial emulation is more appropriate when eligibility or treatment decisions recur over time [14, 15].

### Post-baseline exclusions, incomplete follow-up, and competing events

In observational studies, follow-up should be defined at baseline and proceed according to pre-specified rules, rather than being modified based on information acquired after treatment assignment. Once individuals have entered the study and been assigned to a treatment strategy, they should not be excluded simply because the post-baseline data are incomplete, they deviate from the expected clinical course, or because an intermediate event occurs [5]. If post-baseline deviations are relevant to the estimand, they should be handled based on pre-specified analytic rules, such as censoring with appropriate adjustment for informative censoring.

This issue is particularly relevant in older orthopaedic populations because death, transfer to another facility, incomplete follow-up, and institutionalisation are common and prognostically informative in such populations [16]. For example, in a study of two year revision surgery after THA versus BHA for hip fractures, excluding patients who die early or lack complete follow-up may preferentially eliminate frailer individuals from one treatment group and distort the estimated comparative risk of revision surgery. These issues should be anticipated at the design stage and addressed in a manner consistent with the estimand of interest.

## How to specify and emulate a target trial in orthopaedic research

When applying this framework to routinely collected data, investigators should first specify the protocol of the pragmatic target trial that would ideally answer the causal question, and then describe how that trial would be emulated using the available observational data [3]. If a randomised controlled trial addressing a closely related clinical question already exists, its protocol can provide a useful reference for specifying the target trial.

For clarity and transparency, the target trial and its emulation using observational data should be presented side by side using the core components, viz. eligibility criteria, treatment strategies, treatment assignment, outcomes, start and end of follow-up, causal contrast, and statistical analysis [4]. The first six components define the causal estimand, whereas the statistical analysis specifies the estimator used to estimate it. Figure 2 provides a schematic overview of this process.

To illustrate this process, Table 1 presents a pragmatic target trial and its observational emulation for comparing two year revision surgery after THA versus BHA for hip fracture using a large database of healthcare claims [7]. This example assumes that validation studies have investigated the diagnostic and procedure codes used in this database. The exact definitions should be adapted according to the data source, clinical setting, and estimand of interest. To support practical application, Table 2 provides a self-checklist for investigators designing target trial emulations in orthopaedic surgical research.

**Table 2:** Self-checklist for target trial emulation in orthopaedic observational research, illustrated by THA versus BHA after hip fracture

Domain	General self-check question	Applied example: THA versus BHA after hip fracture
Clinical question	Is the study built around a clearly defined clinical question comparing well-defined treatment strategies in a specified target population?	Is the clinical question framed as a comparison of initial THA versus initial BHA in a specified population of patients with hip fracture, with fracture type clearly defined and with both procedures representing realistic treatment options?
Causal estimand	Has the causal estimand been defined before choosing the estimator?	Is it clear whether the study aims to estimate the effect of assignment to an initial THA strategy versus an initial BHA strategy on 2-year revision surgery in the target population?
Target trial specification and data mapping	Has the protocol of a pragmatic target trial been specified, and can its main components be mapped to the available observational data?	Are the eligibility criteria, treatment strategies, treatment assignment, outcomes, follow-up start and end, causal contrast of interest, statistical analysis, and identifying assumptions specified for a pragmatic target trial comparing THA with BHA? Can hip fracture diagnosis, fracture type, arthroplasty type, procedure date, revision surgery, death, follow-up, and baseline covariates be identified in the database?
Eligibility criteria — target population	Are the eligible individuals those for whom each strategy under comparison would be clinically plausible, with sufficient observed use of both strategies across relevant pretreatment strata?	Are patients restricted to those who could reasonably undergo either THA or BHA, rather than including patients for whom one procedure would not be a realistic option? Is there sufficient overlap in THA and BHA use across clinically relevant pretreatment subgroups, such as those defined by frailty, pre-fracture mobility, cognitive status, and comorbidity?
Eligibility criteria — timing	Are the inclusion and exclusion criteria assessed using only information available at or before time zero?	Are the inclusion criteria, such as age, hip fracture diagnosis, fracture type, and candidacy for hip arthroplasty, and the exclusion criteria, such as previous ipsilateral hip arthroplasty, major trauma, pathological fracture, contraindications to either procedure, severe cognitive impairment, or transfer status, defined without using information that becomes available after time zero?
Treatment strategies	Are the interventions described as treatment strategies, rather than merely as exposure labels?	Are THA and BHA defined as initial surgical strategies, and is it clear whether the surgical approach, implant fixation method, implant design, surgeon experience, hospital surgical volume, and perioperative care are treatment-defining components, usual-care variation, covariates, or unmeasured limitations?
Treatment assignment and time zero	Are treatment assignment and the start of follow-up anchored to the same baseline time?	Are patients classified according to the initial THA or BHA strategy at time zero, rather than according to later revision, complications, transfer, or survival?
Confounding control	Are pretreatment factors associated with both treatment selection and outcomes identified and addressed in the design or analysis, without adjusting for variables measured after time zero as baseline confounders?	Are pretreatment factors that may influence both treatment selection and postoperative outcomes identified and addressed in the design or analysis, including, for example, age, sex, comorbidities, fracture type, pre-fracture mobility, cognitive status, functional status or activities of daily living, pre-fracture residence, medication use, calendar time, hospital type, hospital surgical volume, and other hospital-level factors? Are variables measured after time zero, including, for example, early postoperative complications, postoperative rehabilitation, post-surgical transfer, and care processes influenced by the initial surgical strategy, excluded from the baseline confounder set?
Outcome definition	Is the outcome clinically meaningful and consistently measurable in the data source?	Is 2-year revision surgery defined using clinically interpretable procedure categories, such as component exchange, conversion to THA, implant removal, or reoperation for infection or dislocation, and can these procedures be reliably identified in the database?
Follow-up and post-baseline events	Are the start and end of follow-up, censoring rules, and handling of post-baseline events specified in advance and applied consistently with the estimand?	Does follow-up start at time zero and end according to pre-specified rules, such as at 2 years, loss of database eligibility, the administrative end of the study period, or another prespecified endpoint? Are patients retained according to these rules rather than excluded because of transfer, incomplete claims follow-up, or deviation from the expected postoperative course? If death before revision is relevant, is its handling specified in relation to the estimand?
Causal contrast of interest	Is the causal contrast specified, such as an intention-to-treat effect or a per-protocol effect?	Is the study intended to estimate an intention-to-treat analogue, such as the effect of assignment to an initial THA strategy versus assignment to an initial BHA strategy at time zero among eligible patients with hip fracture?
Statistical analysis	Does the statistical analysis specify an estimator that is aligned with the causal estimand and causal contrast of interest?	Is the planned analysis aligned with the specified contrast? For an intention-to-treat analogue, are patients classified and analysed according to the initial THA or BHA strategy defined at time zero, without subsequent reclassification or exclusion based on post-baseline events?
Identifying assumptions	Are the assumptions required for a causal interpretation stated explicitly and distinguished from the target trial protocol components and the estimator?	For the THA versus BHA comparison, are consistency, conditional exchangeability, and positivity explicitly stated, and are any additional assumptions required for censoring, loss to follow-up, or the prespecified handling of death before revision described?
Measurement validity and data limitations	Are key data elements measured with sufficient validity and completeness, and are important unmeasured or poorly measured variables acknowledged as limitations?	Are the diagnosis codes, procedure codes, procedure dates, revision procedures, death information, hospital identifiers, and follow-up information sufficiently valid and complete for the emulation? Are important variables that are unavailable or poorly measured, such as frailty, pre-fracture mobility, cognitive status, surgeon volume, implant details, or operative approach, acknowledged as limitations?

*THA* total hip arthroplasty, *BHA* bipolar hemiarthroplasty

As observational data do not include random treatment assignments, investigators should also state the assumptions required for the emulation to support a causal interpretation [17]. Table 1 lists separate components of these identifying assumptions. Depending on the estimand and study design, these assumptions may include conditional exchangeability, positivity, consistency, and assumptions related to censoring and prespecified handling of competing events [5].

### Eligibility criteria

In the target trial emulation, the eligibility criteria should define the clinical population for whom each intervention strategy would be a realistic option in routine practice. Therefore, the target population and clinical time points at which individuals become eligible for inclusion should be clarified [4, 5]. This step is essential for aligning the estimand with the clinical question and reducing the positivity violation that arises when a treatment strategy is rarely or never used in certain patient groups.

To preserve the temporal logic of a trial, eligibility should be determined using only the information available up to the time of treatment assignment and not the information acquired thereafter.

When comparing THA versus BHA for hip fracture, time zero may be defined as the date of the index arthroplasty, provided that the target trial is explicitly limited to patients who are candidates for, and proceed to, hip arthroplasty [8].

### Treatment strategies

Treatment strategies should be explicitly defined, because their specification determines the estimated causal effect. A clear specification is necessary to support the consistency assumption [5]. The treatment strategy should be defined with sufficient detail to avoid unintentionally combining clinically important versions of the intervention. However, in orthopaedic research this does not mean that every technical detail of surgical care must be standardised. Some variation may be appropriate when the aim is to estimate the effect of treatment strategies used in routine practice.

Therefore, investigators should specify which elements define the treatment strategy and which are allowed to vary as part of usual care. When important procedural details are unavailable, this limitation should be explicitly described in the emulation, and sensitivity analyses can assess the impact of alternative treatment definitions.

For example, while comparing BHA versus THA for hip fracture, the treatment strategy may be defined primarily by the initial assignment to THA or BHA. Investigators should pre-specify how procedural versions and care-delivery factors, including the surgical approach, fixation method, implant design, perioperative care, hospital volume, and institutional care pathways, will be handled in the emulation, i.e., as treatment-defining components, permissible usual-care variation, baseline or contextual covariates, or limitations when unavailable [7, 11, 18].

### Treatment assignment procedures

The treatment assignment procedures should specify how individuals are classified into treatment strategies at time zero [4]. For observational data, this classification should be based on the information available when eligibility is assessed, treatment strategies are assigned, and follow-up is initiated. Using events that occur after cohort entry to define treatment assignment can introduce avoidable design bias, particularly when the comparison involves treatment timing [4].

As treatment is not randomly assigned in observational studies, investigators should pre-specify how they will address baseline confounding in treatment assignment [5]. Adjustment should focus on pretreatment variables measured before or at time zero that influence treatment selection or timing and are prognostic for outcomes, because these variables are most relevant to the plausibility of conditional exchangeability. Covariates should be selected in advance, based on previous literature, clinical knowledge, and, when helpful, directed acyclic graphs [19]. Variables measured after time zero, including early postoperative events or care processes affected by the assigned strategy, should not be adjusted for as part of baseline confounding.

When the key pre-treatment determinants of treatment choice, treatment timing, or prognosis are unavailable or poorly measured, matching, weighting, or regression adjustment cannot completely eliminate residual confounding [5]. In such cases, investigators should explicitly state this limitation and consider complementary analyses, such as quantitative bias analysis, negative control, or instrumental variable analyses, when a clinically credible instrument exists [8, 20].

### Follow-up commencement and cessation

The start and end of follow-up should be explicitly defined. Once individuals have been assigned to a treatment strategy at time zero, follow-up should proceed according to pre-specified rules [4]. It should not be modified based on the information observed after treatment assignment.

The end of the follow-up period should include clearly defined censoring rules such as an administrative end to the follow-up period, documented loss to follow-up, or predefined protocol deviations. Individuals should not be excluded after baseline simply because their post-baseline data are incomplete or because they deviate from the expected clinical course. Instead, they should be handled using pre-specified rules aligned with the causal question. Examples include treating post-baseline events as censoring events when they are consistent with the target estimand, analysing cumulative incidence in the presence of competing events, or redefining the outcome as a composite endpoint [21]. When treatment strategies involve protocol deviations or treatment changes after baseline, the censoring approach should be aligned with the causal contrast of interest and supported by explicit assumptions and appropriate adjustments.

When studying long-term postoperative outcomes, such as two year revision surgery, in older orthopaedic populations, death can preclude subsequent revision surgery, necessitating explicit consideration in the choice of estimand. Investigators should pre-specify how death will be handled in relation to the estimand using one of the following strategies: estimating the cumulative incidence of revision, with death treated as a competing event, defining a composite outcome such as revision or death, or estimating revision risk under a hypothetical scenario in which death is handled as censoring under explicit assumptions [21].

When treatment strategies depend on the actions that occur after cohort entry, a simple comparison based on the treatment eventually received may misalign eligibility, treatment assignment, and outcome follow-up. This problem is common in studies on treatment timing, such as postoperative complications and mortality after hip fracture surgery performed within 24 h versus after 24 h. In such studies, time zero should be defined as the point at which patients first become eligible for the timing strategies such as hospital admission for hip fractures, rather than the time of surgery. Investigators should consider designs that account for treatment timing, including clone-censor-weighting for strategies assigned at time zero and sequential target trial emulation when eligibility or treatment decisions recur over time [14, 15].

### Causal contrast of interest

The causal contrast of interest specifies the effect to be estimated, such as the intention-to-treat or per-protocol effect.

## Conclusion

Orthopaedic observational research should be designed to answer a categorical clinical question grounded in treatment decisions. Target trial emulation offers a practical way to achieve this by requiring investigators to specify the target trial before analysis and assess whether routinely collected data can credibly support its emulation. This process can reduce avoidable design problems including poorly defined eligibility criteria, ambiguous treatment strategies, misaligned time zero, and inappropriate handling of follow-up. Appropriately used target trial emulation can help orthopaedic researchers produce observational evidence that is more explicitly specified, clinically interpretable, and better aligned with treatment decisions in routine practice.

## Data Availability

No datasets were generated or analysed during the current study.
